# Mesoporous Whitlockite: Synthesis, characterization, and in vitro biocompatibility for bone tissue engineering applications

**DOI:** 10.1016/j.jobcr.2025.09.002

**Published:** 2025-09-03

**Authors:** J.S. Sharukh, Sinduja Palati, Saravanan Sekaran, Dhanraj Ganapathy

**Affiliations:** a Department of Pathology, Saveetha Dental College and Hospitals, Saveetha Institute of Medical and Technical Sciences, Saveetha University, Chennai, 600077, India; b Department of Oral Pathology, Saveetha Dental College and Hospitals, Saveetha Institute of Medical and Technical Sciences, Saveetha University, Chennai, 600077, India; c Department of Prosthodontics, Saveetha Dental College and Hospitals, Saveetha Institute of Medical and Technical Sciences, Saveetha University, Chennai, 600077, India

**Keywords:** Whitlockite, Mesoporous biomaterials, Calcium phosphate, BET analysis, Bone tissue engineering, Cytocompatibility

## Abstract

**Background:**

Whitlockite (WH) is a magnesium-containing calcium phosphate mineral that occurs naturally in bone and teeth. Its biological relevance lies in its ability to promote osteogenesis and provide mechanical stability, making it a strong candidate for bone repair applications. Introducing mesoporosity into Whitlockite is expected to further enhance its biological activity by increasing surface roughness and surface area, which improves protein adsorption and supports cell growth.

**Objective:**

This work focused on preparing mesoporous Whitlockite (Meso-Wh) through a controlled acid treatment method, followed by detailed structural and surface characterization, and an in vitro evaluation of its cytocompatibility.

**Methods:**

Whitlockite was synthesized using a precipitation–hydrothermal method with calcium nitrate, magnesium nitrate, and diammonium hydrogen phosphate precursors. Mesoporosity was induced by hydrochloric acid treatment (pH 4). The particles were characterized using scanning electron microscopy (SEM) with energy-dispersive spectroscopy (EDS), X-ray diffraction (XRD), Fourier transform infrared spectroscopy (FTIR), and nitrogen adsorption–desorption studies with BET and BJH analysis. Cytocompatibility was tested by an indirect MTT assay using MG-63 osteoblast-like cells.

**Results:**

SEM images showed that Meso-Wh particles were smaller and rougher compared with untreated Whitlockite. EDS confirmed calcium, phosphorus, oxygen, and magnesium as the major elements. XRD patterns indicated reduced crystallinity in Meso-Wh, and FTIR spectra revealed broadening of phosphate bands, suggesting lattice disorder due to acid treatment. BET analysis gave a surface area of 63.07 m^2^/g, while BJH pore distribution confirmed mesopores mainly in the 2–5 nm range. MTT results showed good cytocompatibility, with high cell viability at 25–75 % extract dilutions and a slight decrease at 100 %. The positive control exhibited marked cytotoxicity.

**Conclusion:**

Acid treatment effectively produced mesoporous Whitlockite with enhanced surface area and nanoscale porosity, without altering its chemical composition, indicating its suitability for further development as a bone tissue engineering scaffold.

## 1 Introduction

Whitlockite (WH), a calcium phosphate mineral, holds significant promise in biomedical applications, particularly in bone regeneration, due to its remarkable biocompatibility and bioactivity. It is the second most abundant inorganic constituent of bones and teeth, after hydroxyapatite, and contributes to these tissues' mechanical strength and biological function.^1^^,^^2^ Unlike hydroxyapatite (HAP), whitlockite contains magnesium ions, which are known to enhance osteogenesis and promote bone healing processes. Magnesium enhances osteogenesis, making Whitlockite a more effective promoter of bone healing and regeneration.^3^^,^^4^ Research has shown that magnesium-stabilized Ca_3_(PO_4_)_2_ is an osteoconductive and bioresorbable ceramic, making it an ideal candidate for biomaterial applications in bone healing.^5^^,^^6^ Compared to β-TCP, magnesium-containing Whitlockite elicits a more robust osteoconductive response, attributed to the mechanical properties achieved through optimized sintering, closely mimicking those of natural bone.^7^, ^8^, ^9^ This unique composition makes whitlockite an attractive material for developing biomaterials for bone tissue engineering.

The introduction of mesoporous structures to Whitlockite represents an innovative approach to further improve its utility in bone tissue engineering. By increasing the material's surface area and porosity, mesoporous Whitlockite (M − WH) is expected to offer superior osteoconductivity and bioactivity, providing a more favorable environment for bone cell attachment, differentiation, and growth. These features are crucial for biomedical applications as they enhance the material's ability to deliver drugs, proteins, or growth factors, and promote cell attachment and proliferation.^10^, ^11^, ^12^ Moreover, the mesoporous structure can act as a reservoir for the sustained delivery of osteogenic factors, offering additional therapeutic benefits for bone regeneration.^13^

Mesoporous hydroxyapatite, for instance, has been studied for its potential to mimic the natural bone matrix, as it not only facilitates osteogenesis but also serves as a reservoir for the controlled release of drugs, proteins, and growth factors. The mesoporous structure of HAP provides an enhanced surface area that fosters greater interaction with bone cells, promoting faster and more efficient bone regeneration. Studies have demonstrated that mesoporous HAP scaffolds significantly improve bone formation when used in vivo, primarily due to their ability to modulate the local biochemical environment by releasing osteogenic factors like bone morphogenetic proteins (BMPs) in a controlled manner.^14^, ^15^, ^16^ Mesoporous chitosan scaffolds are particularly advantageous for drug delivery applications, as they offer controlled release kinetics and bioactivity. When incorporated with other materials such as hydroxyapatite or bioactive glass, mesoporous chitosan-based scaffolds demonstrate enhanced bone regeneration potential by providing a supportive matrix for cell proliferation and mineralization.^17^ Additionally, the mesoporous nature of these scaffolds facilitates greater nutrient exchange and waste removal, which is critical for successfully integrating the scaffold with host tissue.

To date, no studies have been reported on the synthesis or characterization of mesoporous Whitlockite. This study introduces a novel approach to enhancing the bioactivity of whitlockite by creating mesoporous structures. Characterizing these mesoporous particles includes thoroughly analyzing their morphology, porosity, surface area, in vitro biocompatibility, and osteogenic potential.

## 2 Material and methods

### 2.1 Preparation of Whitlockite (WH)

To synthesize Whitlockite (WH), the synthesis protocol for Whitlockite was adapted and modified from the method described by Ali and Murugan in their work on Whitlockite nanostructures for hemostatic applications.^18^ We prepared several precursor solutions. First, 2.96 g of calcium nitrate tetrahydrate (Ca(NO_3_)_2_·4H_2_O) were accurately weighed and dissolved in 25 mL of distilled water under constant stirring to ensure complete dissolution. In parallel, 0.449 g of magnesium nitrate hexahydrate (Mg(NO_3_)_2_·6H_2_O) were dissolved in 3.5 mL of distilled water, also with thorough stirring. Once both solutions were fully dissolved and homogeneous, they were set aside. Next, 2.5641 g of diammonium hydrogen phosphate ((NH_4_)_2_HPO_4_) were weighed and dissolved in 20 mL of distilled water. This solution was stirred vigorously to achieve complete dissolution. Subsequently, the calcium nitrate and magnesium nitrate solutions were slowly added dropwise to the diammonium hydrogen phosphate solution under continuous stirring to ensure even distribution and homogeneous mixing of all reactants. The pH of the resulting mixture was carefully adjusted to 6 using an ammonia solution (NH_4_OH), added dropwise while monitoring with a pH meter. Once the pH was stabilized at 6, the solution was stirred for an additional 60 min to allow the reaction to proceed. The mixture was then autoclaved at 200 °C for 15 min to induce crystallization and form the calcium-magnesium phosphate composite. After autoclaving, the mixture was cooled to room temperature. To remove any unreacted substances and impurities, the precipitate was thoroughly washed with ethanol multiple times, followed by filtration. The cleaned precipitate was dried overnight at 100 °C, yielding the final calcium-magnesium phosphate composite – Whitlockite (WH) in powder form.

### 2.2 Synthesis of Mesoporous Whitlockite (M − WH)

To create mesoporous Whitlockite (M − WH), we began by taking 0.5 g of the previously prepared Whitlockite (WH) powder and dispersing it in 10 mL of distilled water. This dispersion was stirred for 15 min to ensure that the WH powder was evenly suspended in the solution. Following this, hydrochloric acid (HCl) was added dropwise to the suspension, carefully monitoring the pH during the process. The pH gradually lowered to 4, ensuring that the acid was added slowly and uniformly to avoid rapid changes in the mixture. Once the desired pH was reached, the solution was stirred continuously for 60 min to allow complete interaction between the WH particles and the acidic medium, facilitating the formation of a mesoporous structure. After stirring, the mixture was subjected to drying at 100 °C to remove excess water and obtain the mesoporous WH powder. The resulting powder was carefully collected and stored for further characterization and in vitro testing.

### 2.3 Characterization of mesoporous whitlockite particles

The morphology, size, and surface composition of meso-Wh particles were investigated using scanning electron microscopy (SEM) equipped with energy dispersive spectroscopy (EDS). For SEM imaging (HR-SEM Quanta 200 FEG, The Netherlands), particles were first sonicated, mounted on glass coverslips, and sputter-coated with gold. Micrographs were obtained at accelerating voltages of 10–15 kV across various magnifications. Fourier transform infrared (FTIR) spectroscopy (PerkinElmer, USA) was employed to identify functional groups and confirm bonding characteristics. Spectra were recorded in the wavenumber range of 4000–450 cm^−1^ using the KBr pellet method.

Phase purity and crystallinity were analyzed by X-ray diffraction (XRD, PANalytical X'Pert PRO, The Netherlands) operated at 40 kV using Cu Kα radiation (λ = 1.5406 Å). Diffraction patterns were collected at room temperature over the 2θ range of 10°–70°, with a scan rate of 2°/min, and peak indexing was performed with reference to JCPDS standard data for whitlockite.

Textural properties were determined by nitrogen adsorption–desorption analysis (ASAP 2020, Micromeritics, USA). Prior to measurements, samples were degassed under vacuum at 200 °C for 5 h. Nitrogen adsorption was carried out at 77.3 K. The Brunauer–Emmett–Teller (BET) method was applied in the relative pressure range (P/P_0_) of 0.05–0.33 to estimate surface area, while mesopore size distribution was calculated using the Barrett–Joyner–Halenda (BJH) model from the desorption isotherm branch.

### 2.4 Cell culture

The human osteoblast-like cell line MG-63 was obtained from the National Centre for Cell Sciences (NCCS, Pune, India). Cells were routinely maintained in Dulbecco's Modified Eagle Medium (DMEM, high glucose formulation) supplemented with 10 % heat-inactivated fetal bovine serum (FBS; Gibco, USA), 100 U/mL penicillin, and 100 μg/mL streptomycin. Cultures were incubated under standard conditions at 37 °C in a humidified atmosphere containing 5 % CO_2_. The medium was replenished every 2–3 days to maintain optimum nutrient balance and prevent accumulation of metabolic by-products.

Once cultures reached approximately 80–90 % confluence, the adherent cells were enzymatically detached using 0.25 % trypsin–EDTA solution. After neutralization with complete medium, the cell suspension was centrifuged at 1000 rpm for 5 min, resuspended in fresh culture medium, and seeded into new flasks at appropriate split ratios for expansion. The cells were serially passaged and only those within passages 5–15 were used for subsequent experimental procedures to ensure reproducibility and to avoid phenotypic drift.

### 2.5 Biocompatibility assessment by indirect MTT assay

The cytocompatibility of meso-Wh (m-WH) particles was assessed using an indirect extract-based MTT assay in accordance with ISO 10993–6:2016 guidelines, with extraction and cytotoxicity testing principles adapted from ISO 10993-5 and ISO 10993-12. Sterilized m-WH particles were incubated in complete Dulbecco's Modified Eagle Medium (DMEM supplemented with 10 % fetal bovine serum, 100 U/mL penicillin, and 100 μg/mL streptomycin) at a ratio of 0.1 g/mL under static conditions at 37 °C for 24 h. The resulting supernatant (extract) was clarified by centrifugation and filtered through a 0.22 μm syringe filter. Negative control extracts were prepared from high-density polyethylene, while phenol-containing medium (0.64 %) served as the positive control.

For MTT asssay, cells between passages 5 and 15 were seeded in 96-well plates at a density of 1 × 10^4^ cells/well and allowed to adhere for 24 h. Culture medium was replaced with 100 μL of test extracts, either undiluted (100 %) or serially diluted (100 %, 75 %, 50 %, and 25 %) in complete medium. Cells without treated as control and cells exposed to 0.1 % Triton-X-100 served as positive control. Following 24 h incubation at 37 °C, cell viability was determined. After extract exposure, 10 μL of MTT reagent (5 mg/mL stock in PBS, final concentration 0.5 mg/mL) was added to each well and incubated for 4 h. The supernatant was carefully removed, and the purple formazan crystals formed by metabolically active cells were dissolved in 100 μL dimethyl sulfoxide (DMSO). Absorbance was measured at 570 nm with a reference wavelength of 630 nm using a microplate reader (BioTek, USA).

### 2.6 Statistical analysis

All experiments were conducted in triplicate (n = 3), and results are expressed as mean ± standard deviation (SD). Statistical analysis was performed using the Student's t-test to compare differences between treated groups and the control. A p value of less than 0.05 was considered statistically significant.

## 3 Results

### 3.1 Morphological characterization of the mesoporous whitlockite particles

**Fig. 1:**
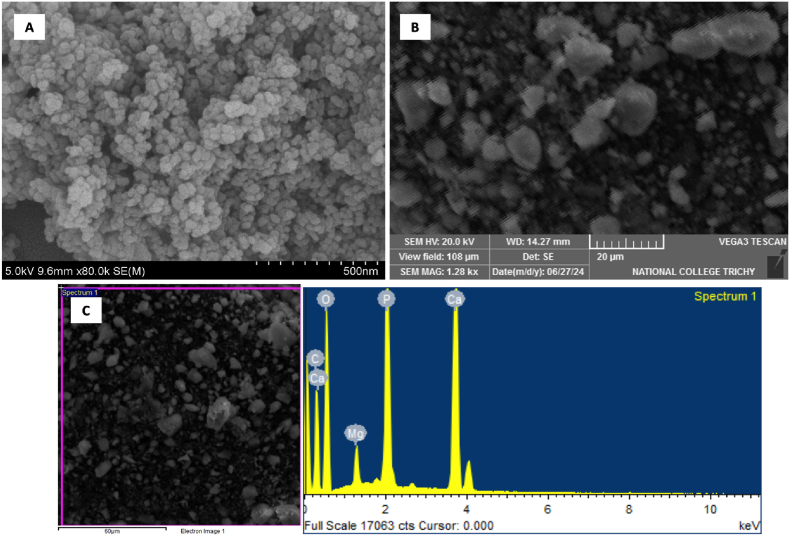
Structural and compositional features of Whitlockite and mesoporous Whitlockite (M − WH). (A) High-magnification SEM image (80,000X) showing nano-sized whitlockite particles. (B) SEM image (1.28kX) depicting aggregated clusters of mesoporous whitlockite particles with roughened morphology. (C) SEM micrograph (left) and the corresponding EDS spectrum (right) highlighting the elemental composition of M-WH. Peaks corresponding to calcium (Ca), phosphorus (P), oxygen (O), and magnesium (Mg) were observed, consistent with the expected structure of Whitlockite. A minor carbon (C) peak was also present, which may originate from surface adsorption or sample handling.

The Scanning Electron Microscope (SEM) images (Fig. 1A and B) highlight the distinct morphological characteristics of WH and M-WH. In the case of WH, the images reveal relatively larger, well-defined crystalline particles, accompanied by clusters of irregularly shaped particles with a rough and porous surface texture. Such morphological features are typical for calcium phosphate biomaterials in their raw crystalline form. In contrast, the SEM images of M − WH show smaller, irregularly shaped particles with a more pronounced roughness. Across CaP systems, acid treatments are routinely used to increase nanoscale roughness/porosity and reactivity. Even in demineralized bone-derived CaP, mild acidification increases surface roughness and microtopography, consistent with a dissolution-led origin of meso- and nano-porosity. The Energy Dispersive Spectroscopy (EDS) analysis reveals distinct peaks corresponding to calcium (Ca), phosphorus (P), and oxygen (O), which are the fundamental constituents of Whitlockite (Fig. 1C). These elements confirm the expected chemical composition of Whitlockite as Ca_9_Mg(HPO_4_)(PO_4_)_6_.

### 3.2 Reduction in crystallinity and structural order following acid modification

**Fig. 2:**
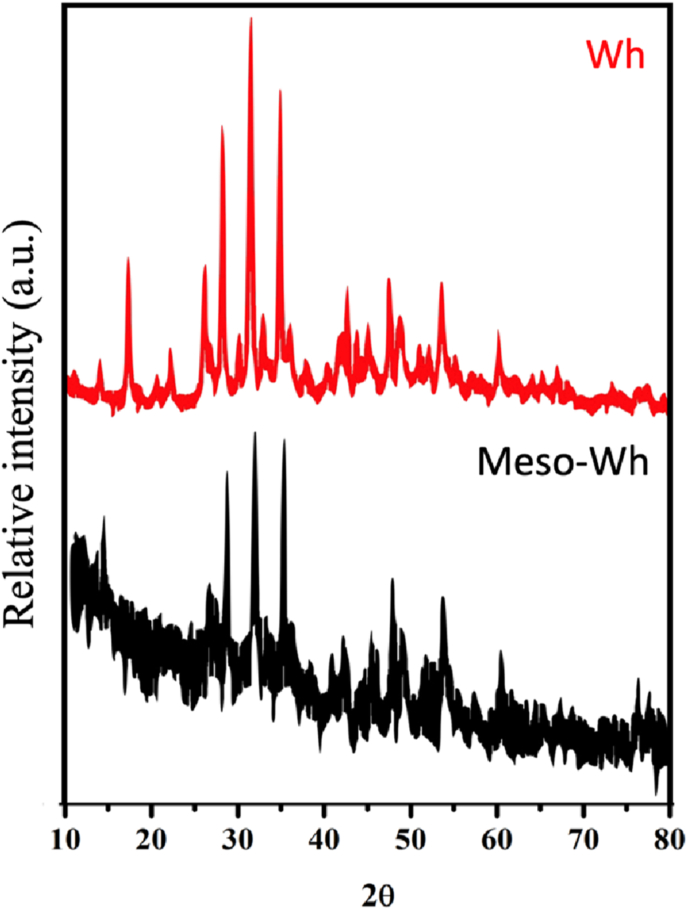
X-ray diffraction (XRD) patterns of Whitlockite (Wh) and mesoporous Whitlockite (Meso-Wh). The pristine Whitlockite sample (red) exhibited sharp and well-defined diffraction peaks corresponding to crystalline calcium–magnesium phosphate. In contrast, the mesoporous Whitlockite (black) showed broader peaks with reduced intensity, indicating a partial loss of crystallinity and increased structural disorder after acid treatment. This reduction in crystallinity is consistent with the introduction of mesoporosity and surface modifications. (For interpretation of the references to colour in this figure legend, the reader is referred to the Web version of this article.)

X-ray diffraction (XRD) patterns (Fig. 2) for both WH and Meso-WH indicate the presence of characteristic peaks corresponding to highly crystalline phases. In the case of WH, sharp and intense diffraction peaks confirm the material's high crystallinity, with diffraction data closely matching those of magnesium-doped Whitlockite as per the Joint Committee on Powder Diffraction Standards (JCPDS) reference (ICDD #04-009-3397). These crystalline peaks are indicative of the stable bioceramic phase of Whitlockite, essential for its structural integrity and potential as a bone substitute material. In contrast, the XRD pattern of Meso-WH displays broader and less intense peaks, suggesting a reduction in crystallinity compared to WH. This decrease in crystallinity is expected due to the introduction of mesoporous structures, which disrupt the regular crystal lattice. Importantly, the lack of diffraction peaks corresponding to secondary phases confirms the high purity of the synthesized mesoporous Whitlockite, ensuring that the modifications during synthesis did not result in unwanted by-products.

**Fig. 3:**
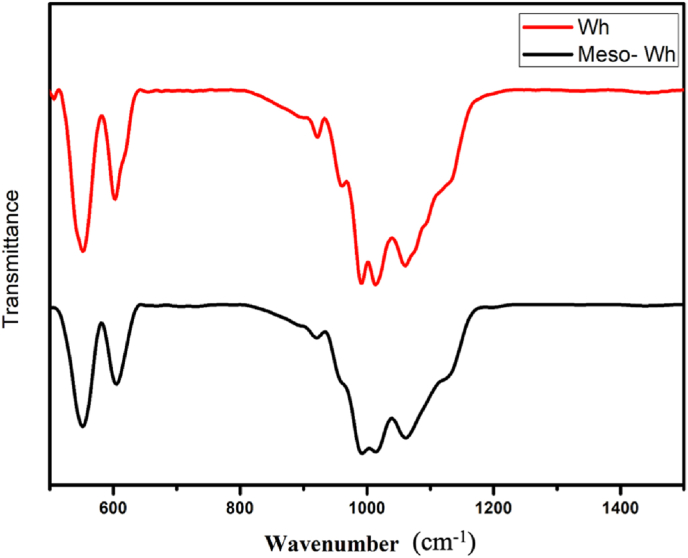
Fourier transform infrared (FTIR) spectra of Whitlockite (Wh, red) and mesoporous Whitlockite (Meso-Wh, black). Both samples exhibited characteristic phosphate (PO_4_^3−^) vibrational bands in the region of 500–1200 cm^−1^, confirming the phosphate framework of Whitlockite. In the mesoporous sample, these peaks appeared slightly broadened and shifted compared to pristine Whitlockite, reflecting structural modifications. (For interpretation of the references to colour in this figure legend, the reader is referred to the Web version of this article.)

Fourier Transform Infrared Spectroscopy (FTIR) spectra for both WH and M − WH (Fig. 3) display characteristic vibrational bands associated with phosphate (PO_4_^3−^) groups. In WH, strong absorption bands around 560 cm^−1^ and 1020 cm^−1^ correspond to phosphate stretching and bending modes, affirming the material's phosphate-based structure. A prominent peak at approximately 1060.26 cm^−1^ is attributed to the antisymmetric stretching vibration of the phosphate group, consistent with the crystalline structure of Whitlockite. The smaller peaks associated with carbonate groups are also visible in the spectra. A secondary antisymmetric stretching vibration is responsible for another peak at 1013.51 cm^−1^. The peak at 991.27 cm^−1^ represents the symmetric stretching vibration of the phosphate group, which corresponds within the typical range reported for Whitlockite. In M − WH, these vibrational bands exhibit slight shifts and broadening, particularly around 560 cm^−1^ and 1020 cm^−1^, indicative of the increased surface area and porosity due to the mesoporous structure. When compared with Whitlockite, the spectrum of mesoporous Whitlockite displayed noticeable broadening and a slight shift in the intensity of these phosphate bands. Such changes suggest a reduction in structural order and crystallinity following the acid treatment, consistent with XRD results. Importantly, no new peaks were introduced, indicating that the fundamental chemical composition of Whitlockite remained intact. The subtle spectral modifications reflect lattice distortions and surface disorder associated with the development of mesoporous architecture.

### 3.3 Acid treatment introduced mesoporosity and increased the surface area in WH particles

**Fig. 4:**
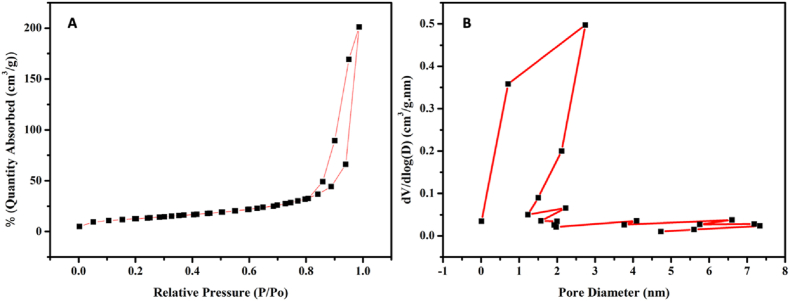
Nitrogen adsorption–desorption isotherms and pore size distribution of mesoporous Whitlockite (Meso-Wh). (A) Adsorption–desorption isotherms demonstrating type IV behavior with H3 hysteresis loops, characteristic of mesoporous materials. Meso-Wh showed slightly higher adsorption at relative pressures above 0.8, suggesting the development of mesoporosity. (B) BJH pore size distribution curves illustrating a shift toward smaller pore diameters in Meso-Wh. The results confirm that acid treatment successfully modified the textural properties of Whitlockite, producing additional mesopores in the 2–5 nm range.

The nitrogen adsorption–desorption isotherm of mesoporous Whitlockite (Meso-Wh) exhibited a type IV curve with a distinct H3 hysteresis loop at higher relative pressures (Fig. 4). This adsorption profile is a characteristic feature of mesoporous materials, confirming the successful introduction of mesoporosity into the Whitlockite structure following acid treatment. The steep rise in adsorption volume at relative pressures above 0.8 indicates the presence of capillary condensation within mesopores. Particles synthesized at showed a surface area of 63.072 m^2^/g.

The BJH pore size distribution (Fig. 4B) revealed that the majority of pores were located within the mesoporous range of 2–5 nm, with a sharp peak around ∼2–3 nm. This narrow distribution suggests that the etching process generated relatively uniform mesopores. The presence of these nanoscale pores enhances the surface area and increases the likelihood of protein adsorption and ion exchange, both of which are beneficial for biomedical applications.

Overall, the analysis confirms that Meso-Wh possesses a mesoporous structure with well-defined pore diameters, making it a suitable candidate for use in bone tissue engineering, where high surface reactivity and porosity are desirable for cell–material interactions and osteogenic activity.

### 3.4 Mesoporous Whitlockite particles are Biocompatible

**Fig. 5:**
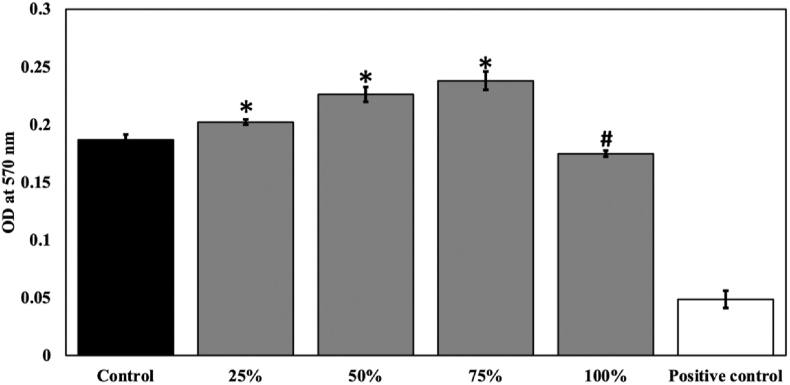
Cytocompatibility assessment of mesoporous Whitlockite (Meso-Wh) extracts at different concentrations assessed by indirect MTT assay on MG-63 cells. The bars represent mean cell viability (%) ± standard deviation (SD) after 24 h exposure to extract dilutions (25 %, 50 %, 75 %, and 100 %) compared with untreated control and positive control. Cell viability remained high across 25–75 % concentrations, with the maximum at 75 %, which was significantly greater than the control (p < 0.001). The 100 % extract showed a modest but statistically significant reduction in viability compared with control (p < 0.05). Cells without any treatment served as negative control and cells exposed to 0.1 % Triton-X-100 served as positive control. The experiments were performed in biological replicates (n = 3). ∗- indicates significant increase compared to the control and #- indicates significant reduction compared to the control.

Any material intended for biomedical applications shouldn't exert The cytocompatibility of mesoporous Whitlockite (Meso-Wh) was examined using an indirect MTT assay on MG-63 osteoblast-like cells. Cell viability was maintained at high levels across the tested extract dilutions of 25 %, 50 %, and 75 %, indicating that the material was well tolerated by the cells (Fig. 5). Among these, the 75 % extract supported the highest cell viability, followed by the 50 % extract, both of which demonstrated favorable cellular responses. The 25 % extract also showed good compatibility, though slightly lower than the other two concentrations. When cells were exposed to the 100 % extract, a mild reduction in viability was observed compared with the lower concentrations, although cell survival remained above acceptable cytocompatibility limits. In contrast, the positive control showed a strong cytotoxic effect, with markedly reduced cell viability. These findings confirm that mesoporous Whitlockite possesses good biocompatibility, with particularly favorable responses at intermediate extract concentrations. The results suggest that the material provides a supportive environment for osteoblast growth and holds promise for use in bone tissue engineering applications.

## 4 Discussion

The results of this study highlight the potential of mesoporous Whitlockite (M-Wh) as a promising biomaterial for bone regeneration applications. Mesoporous materials offer advantages in drug delivery and bone regeneration due to their expansive surface area and customizable pore sizes, which promote molecular mobility and accessibility.^19^, ^20^, ^21^ Treating whitlockite with dilute HCl (pH ≈ 4) produced a clear mesoporous signature—type-IV N_2_ isotherm with H3 hysteresis and a BET surface area of ∼63 m^2^ g^−1^, with BJH pores concentrated at ∼2–5 nm. The mechanism is consistent with proton-promoted dissolution of calcium phosphates: surface PO_4_ groups are protonated (→ HPO_4_^2−^), which facilitates Ca^2+^ (and Mg^2+^) detachment and nucleates nanoscale etch pits that later coalesce into mesopores. Although most mechanistic work was done on apatites, the same acid-etch pathway applies to CaP lattices in general and explains our observations on Mg-stabilized β-TCP/whitlockite. Whitlockite is the Mg-enriched analogue of β-TCP and releases Ca^2+^, PO_4_^3−^, and Mg^2+^ ions—magnesium being particularly relevant for osteogenesis and remodeling. Enhancing surface area via mesoporosity increases solid–liquid interfacial reactivity, accelerating ion exchange and protein adsorption and thereby improving early cell–material interactions.

One of the primary benefits of incorporating a mesoporous structure into Whitlockite is its enhanced bioactivity. Scanning electron microscopy (SEM) images demonstrate that M-Wh has a rougher and more porous surface compared to its non-mesoporous counterpart, which shows a highly aggregated structure aligns with previous literature reports, which describe Whitlockite as exhibiting irregular morphology and dense particle packing, resulting in minimal porosity.^22^^,^^23^ This structural characteristic is vital for promoting cellular activities essential for bone healing and regeneration. Previous studies have highlighted the importance of porosity and surface roughness in augmenting osteoconductivity and the overall regenerative potential of biomaterials.^22^^,^^23^ These porous features make Meso-WH particularly suitable for bone tissue engineering, as the porosity allows for improved integration with biological tissues, thereby promoting cellular attachment, proliferation, and differentiation which are the key factors in bone healing and regeneration. Notably, the presence of magnesium (Mg) is significant, as it plays a critical role in stabilizing the Whitlockite structure and enhancing its mechanical properties. Magnesium's incorporation into the crystal lattice of Whitlockite is known to facilitate osteogenesis and improve the material's bioresorbability.^22^, ^23^, ^24^, ^25^, ^26^, ^27^, ^28^

These properties are critical in biomedical applications, especially for bone tissue engineering, as they enable better cell attachment, proliferation, and nutrient exchange. The results from the MTT assay further affirm the biocompatibility of M-Wh, revealing minimal cytotoxic effects, which is essential for any biomaterial intended for clinical use. XRD analysis revealed significant differences in crystallinity between Whitlockite (WH) and mesoporous Whitlockite. WH exhibited sharp and intense peaks indicative of a highly crystalline structure, while M-Wh displayed broader and less intense peaks, suggesting reduced crystallinity. This decrease in crystallinity, while often associated with diminished mechanical properties, can be advantageous for osteoinductive applications. Lower crystallinity may enhance the material's bioresorption rate and facilitate the remodeling process during bone healing.^29^, ^30^, ^31^

Jang and colleagues 7 conducted comprehensive in vitro and in vivo studies comparing WH nanoparticles with hydroxyapatite (HAP) and β-tricalcium phosphate (β-TCP). Their findings indicated that WH-fabricated cellular scaffolds demonstrated superior differentiation behavior in multipotent cells compared to HAP. Furthermore, WH-based implants exhibited improved bone regeneration in vivo, with moderate resorbability relative to HAP and β-TCP. Consequently, synthetic WH emerges as a superior biomaterial characterized by high bioactivity, making it an excellent candidate for applications in bone repair and tissue engineering.

Future studies should investigate the in vivo osteogenic potential of Meso-WH to validate its efficacy in promoting bone regeneration. Additionally, exploring the controlled release of osteogenic factors from Meso-WH could significantly enhance its therapeutic potential. Understanding the interactions between Meso-WH and various cell types will be essential for optimizing its design for specific biomedical applications. Future work should also focus on comparative biocompatibility and osteoinductive studies of Meso-WH against widely used materials like HAp and β-TCP. Evaluating magnesium ion release and its correlation with osteogenic markers will be critical to validate its bone regeneration potential. These investigations will pave the way for the development of advanced biomaterials, potentially leading to improved patient outcomes in bone repair and regenerative medicine.

## 5 Conclusion

Our study successfully synthesized and characterized mesoporous Whitlockite, demonstrating its enhanced structural, compositional, and functional properties compared to non-porous Whitlockite. The mesoporous structure significantly improves the material's bioactivity, surface area, and biocompatibility, making it a superior candidate for bone tissue engineering applications.

Taken together, these findings suggest that mesoporous Whitlockite combines the intrinsic bioactivity of Whitlockite with the advantages of mesoporous architecture, offering a material that can promote cell attachment, proliferation, and bone regeneration. Future in vivo studies, particularly those evaluating osteogenic differentiation, ion release behavior, and integration with host tissue, will be essential to establish its clinical applicability. By uniting structural versatility with biological safety, M-Wh represents a promising step toward the development of next-generation scaffolds for bone repair and regenerative medicine.

## Patient's/Guardian's consent

NA.

## Ethical clearance

The ethical clearance was received from the Institutional ethics board.

## Sources of funding

NIL.

## Declaration of competing interest

The authors declare that they have no known competing financial interests or personal relationships that could have appeared to influence the work reported in this paper.
